# Malignancy in dermatomyositis: a mono-centric retrospective study of 134 patients in China and a potential predictive model

**DOI:** 10.3389/fmed.2023.1200804

**Published:** 2023-06-08

**Authors:** Zhuang-Li Tang, Chao-cheng Chi, Zhen-Wei Tang, Xia-Wei Li, Xiao-Yong Man

**Affiliations:** ^1^Department of Dermatology, The Second Affiliated Hospital, Zhejiang University School of Medicine, Hangzhou, China; ^2^Department of Gastrointestinal Surgery, The Second Affiliated Hospital, Zhejiang University School of Medicine, Hangzhou, China

**Keywords:** dermatomyositis, malignancies, autoantibody, tumor biomarkers, predictive model, receiver operating characteristic curve

## Abstract

**Objectives:**

To describe the demographics and phenotypes of malignancies-associated dermatomyositis (MADM) in east China and pinpoint potential factors indicative of malignancies in patients with dermatomyositis and establish a predictive model.

**Methods:**

We retrospectively analyzed clinical data from 134 patients with adult-onset dermatomyositis hospitalized between January 2019 and May 2022 in one comprehensive hospital. Clinical data including disease course, initial symptoms and signs, and demographic information were retrieved from the Electronic Medical Records System. Other parameters including myositis-specific autoantibodies profiles, ferritin, sedimentation, etc. were all referable. Multivariable multinomial logistic regression was employed to simulate a model to predict cancer risks. Receiver operating characteristic curve was adopted to evaluate the potency of the model.

**Results:**

134 patients with adult-onset dermatomyositis were aptly enrolled in this study based on inclusive and exclusive criteria: 12 (8.96%) with malignancies, 57 (42.53%) with aberrant tumor biomarkers but no malignancies, 65 (48.51%) with neither malignancies nor abnormal tumor biomarkers. Senior diagnostic age, higher LDH, higher ferritin, positive anti-TIF1γ and anti-Mi2 rather than anti-NXP2 autoantibodies were positive indicators of malignancies. Additionally, neither initial complaints nor signs were found to be correlated to a tendency towards malignancies. Digestive system, nasopharyngeal, and lung malignancies were mostly documented in east China. One multivariable multinomial logistic regression model was established to predict the phenotypes of dermatomyositis on the basis of potential malignancies and the overall sensitivity and specificity was satisfactory.

**Conclusion:**

Positivity of anti-TIF1γ and anti-Mi2 autoantibodies are highly indicative of malignancies while the role of anti-NXP2 autoantibody in MADM in the Chinese population remains unclear. The phenotypes of malignancies can be predicted through the model and the predictive power is sufficient. More attention should be paid to malignancies screening in patients with aberrant tumor biomarkers but no malignancies, particularly digestive system, nasopharyngeal, and lung malignancies in patients with dermatomyositis but without malignancies.

## Highlights

- Anti-TIF1γ and anti-Mi2 autoantibodies rather than anti-NXP2 were proven positively correlated to malignancies in this area.
- Malignancies of the gastrointestinal tract, nasopharynx, and lung ranked highest in the study.
- One powerful multivariable multinomial logistic regression model was established to predict the phenotypes of dermatomyositis.

## Introduction

Dermatomyositis (DM) is an autoimmune disease entity that affects both skin and musculature. According to the criteria of the classification of Bohan and Peter, malignancies-associated DM (MADM) is listed as an isolated subgroup (1). Basically, cancers may occur prior to, along with, or following the initial symptoms and signs of DM (2). Data indicated that 7%–30% of DM patients exhibited an association with malignancies, with most studies clustering around 15%–20% (3). Notably, the risk of malignancies in adult patients with DM was reported to be 4.66-fold higher compared to that in normal individuals (4).

Multiple indexes have been suggested as indicative of developing cancers in DM, including older age, male sex, low complement C4, lymphocytosis, poor response to corticosteroids, and rapid disease progression (5). Also, several myositis-specific autoantibodies (MSAs), including anti-TIF1γ and anti-NXP2, have been reported to be associated with increased risk of cancers among patients with DM (6–8). However, most of the conclusive findings were based on the Caucasian population while real-world evidence of the Chinese population remains quite limited so far (9). Furthermore, quantitative models which can be adopted to predict cancer risks in patients with DM are highly needed in clinical practices.

This article aims to contribute to a more comprehensive understanding of dermatomyositis in regard to malignancies. To our knowledge, most scholars focused mainly on the variance of malignancies in patients with DM; however, patients without malignancies but with aberrant tumor biomarkers have always been omitted in cohort or cross-sectional analysis. Therefore, we categorized all the enrolled patients into three groups according to the existence of malignancies and abnormal tumor biomarkers. Subsequent phenotype depiction, risk factors analyses and model establishment were conducted.

## Materials and methods

### Patients

This work is a retrospective, single-centric data analysis of patients diagnosed with DM that were hospitalized in the Department of Dermatology of the Second Affiliated Hospital of Zhejiang University between January 2019 and May 2022. This work is approved by the Second Affiliated Hospital IRB, Zhejiang University School of Medicine (2022-0867) and verbal informed consent was obtained through telephone. All the records were retrieved through the EMRS.

Eligible patients were enrolled based on the following criteria: (a) highly suspected or definite diagnosis of dermatomyositis (score points ≥ 5.5 without muscle biopsy or score points ≥ 6.7 with muscle biopsy) based on 2017 EULAR/ACR classification criteria for adult and juvenile idiopathic inflammatory myopathies (10) and (b) availability of detailed MSAs and myositis-associated autoantibodies (MAAs) data regardless of the results. Any cases with the diagnosis of either mixed connective tissue disease or suspected dermatomyositis (score points < 5.5 without muscle biopsy or score points < 6.7 with muscle biopsy) were excluded from this study.

**Figure 1:**
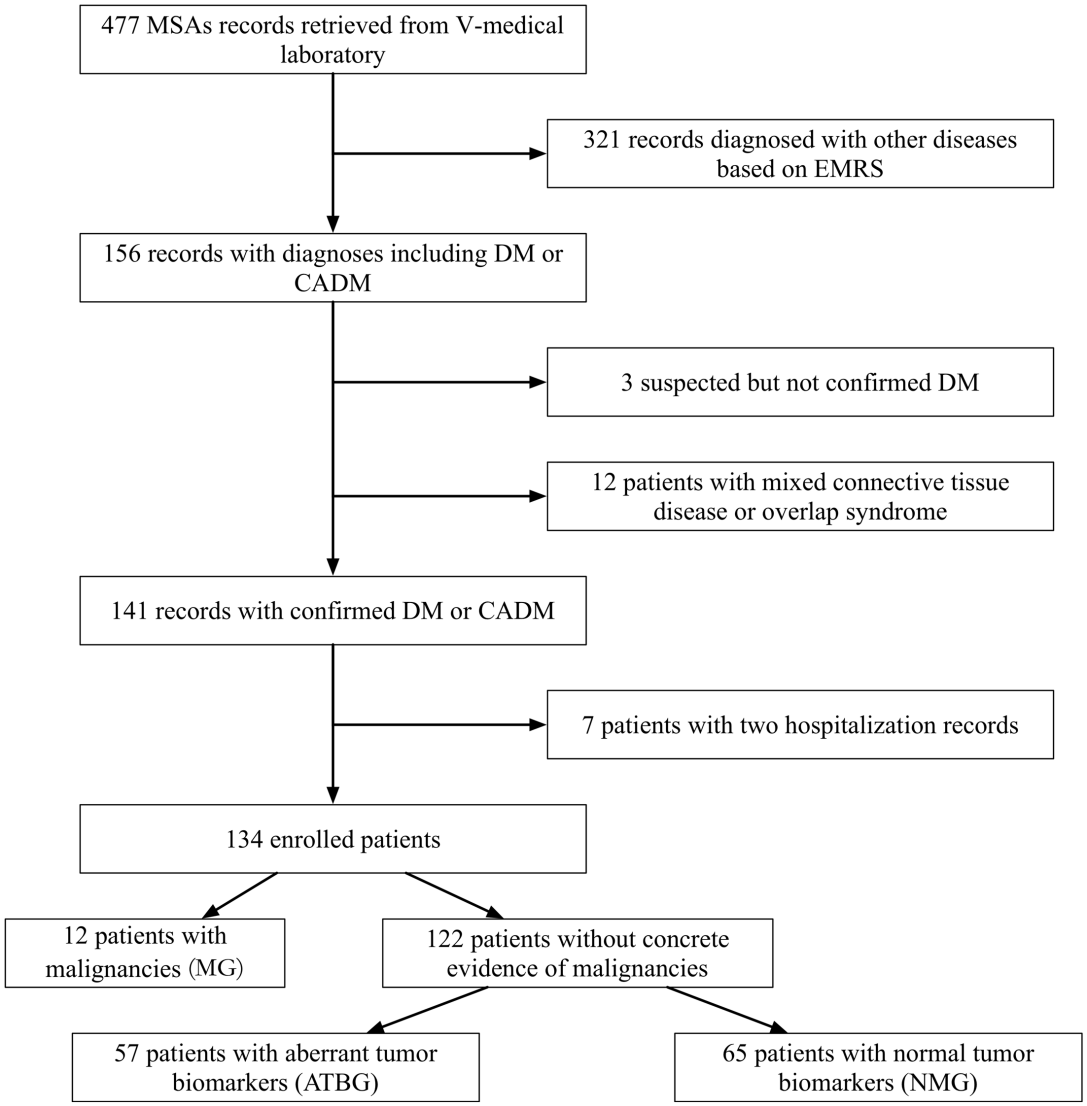
Patient collective flowchart. Initially, 477 records with concrete MSAs and MAAs were documented. Rough screening was conducted owing to diagnoses and 134 patients were duly enrolled. All the enrolled patients were then subdivided into three groups on the basis of malignancies and tumor biomarkers.

Accordingly, 141 of the original 477 records were documented, and 7 identical patients with two records at different times were excluded (Figure 1). The remaining 134 patients with DM were divided into three groups. Patients previously or currently confirmed to have malignancies were categorized into the malignancy group (MG); those with anomalous tumor biomarker results but no validated malignancies were sorted into the aberrant tumor biomarkers group (ATBG); the remaining patients were allocated to the non-malignancy group (NMG).

### Data

Profiles of MSAs and MAAs were detected via the EUROBlotMaster system and the results were retrieved from the V-medical laboratory, Hangzhou. The demographic information including gender, age of diagnosis, disease course, initial symptoms or signs, time gap from initial symptoms to final diagnosis, and laboratory parameters in this study were obtained. Laboratory parameters studied in this research included β2-microglobulin, ferritin, sedimentation, lymphocytes, the neutrophil/lymphocyte ratio (NLR), tumor biomarker profiles, etc. There are few clinical data missing in this study, and the median substitution method is adopted for data processing. Of note, all the validated malignancies were confirmed by specialists from departments of oncologists and pathologists.

### Statistics

The data analysis was performed with the nnet package (version 7.3-18) of R software. A *p* value of <0.05 was considered statistically significant. For independent, homoscedastic, and quantitative data, significance was assessed by ANOVA test. For those data which did not meet homoscedasticity, a non-parametric test was introduced. To assess independent, categorical data, significance was measured by a Chi-square test.

### Model

Multivariable multinomial logistic regression (MLR) was employed to simulate a model to differentiate MG, ATBG, and NMG on the basis of initial clinical parameters upon admission. Furthermore, receiver operating characteristic curve was adopted to evaluate the potency and efficacy of the model.

## Results

### Patient collective

Of the recorded 134 patients (50 males and 84 females) examined, the total prevalence of malignancies was 8.96% (12/134). Apart from that, 57 patients (42.53%) were divided into ATBG, and the remaining 65 patients were allocated to NMG (Figure 1). The gender ratio in each of the three groups was similar without any significant intergroup statistical difference (Table 1).

**Table 1:** Demographic and laboratory parameters among different subgroups.

	MG	ATBG	NMG	*p* value
Gender, *n* (%)				0.887
Male	5 (3.73%)	22 (16.42%)	23 (17.16%)	
Female	7 (5.22%)	35 (26.12%)	42 (31.34%)	
Age of diagnosis (Y, $\bar{x}\pm \delta$ )	59.5 0± 11.82	56.67 ± 11.65	48.18 ± 15.60	0.001^**^
Disease course (D, $\bar{x}\pm \delta$ )	670.08 ± 592.98	798.14 ± 758.95	755.09 ± 572.94	0.777
Time gap from symptoms to diagnosis (M, $\bar{x}\pm \delta$ )	21.92 ± 31.10	20.6 1± 57.25	25.42 ± 55.31	0.286
Clinical parameters ( $\bar{x}\pm \delta$ )				
β2-MG (mg/L)	3.75 ± 2.863	3.0 6± 0.869	2.83 ± 0.523	0.224
ESR (mm/h)	21.67 ± 17.369	37.05 ± 23.33	24.80 ± 25.504	0.0002^**^
LDH (U/L)	433.58 ± 306.13	331.88 ± 230.01	254.68 ± 101.54	0.043^*^
CK (U/L)	586.50 ± 1068.68	775.67 ± 2538.88	282.63 ± 725.02	0.321
CK-MB (U/L)	47.58 ± 59.721	41.75 ± 69.582	26.95 ± 33.710	0.167
Ferritin (μg/L)	412.28 ± 385.85	475.79 ± 962.12	203.44 ± 317.43	0.034^**^
Lymp (×10^9^)	0.82 ± 0.564	1.49 ± 0.829	1.83 ± 0.957	0.0005^**^
NLR	10.15 ± 6.227	11.06 ± 16.872	5.07 ± 5.197	0.0001^**^
MSAs (positive rate)				
Anti-TIF1γ	5/12	5/57	11/65	0.016^*^
Anti-NXP2	0/12	2/57	3/65	0.736
Anti-Mi2	4/12	3/57	5/65	0.007^**^
Anti-MDA5	1/12	21/57	14/65	0.052
Anti-SAE	0/12	2/57	1/65	0.657
Anti-Jo1	0/12	7/57	4/65	0.260
Anti-PL7	0/12	3/57	5/65	0.561
Anti-PL12	0/12	1/57	1/65	0.901
Anti-OJ	0/12	2/57	0/65	0.254
Co-expression of MSAs	2/12	3/57	4/65	0.265

Y, years of life; D, days; M, months; *n*, number; MG, malignancy group; ATBG, aberrant tumor-biomarker group; NMG, non-malignancy group; $\bar{x}$ , mean; δ, standard deviation/*n*, number; β2-MG, β2 microglobulin; ESR, sedimentation; LDH, lactate dehydrogenase. *p value < 0.05; **p value < 0.01.

The mean age of initial diagnosis of dermatomyositis ranked the top in MG followed by that of ATBG and NMG, respectively, with statistical significance (ANOVA test; *p* = 0.001). Nonetheless, no statistical difference was reported in relation to disease course and time gap from symptoms to diagnosis among each group (Table 1).

**Figure 2:**
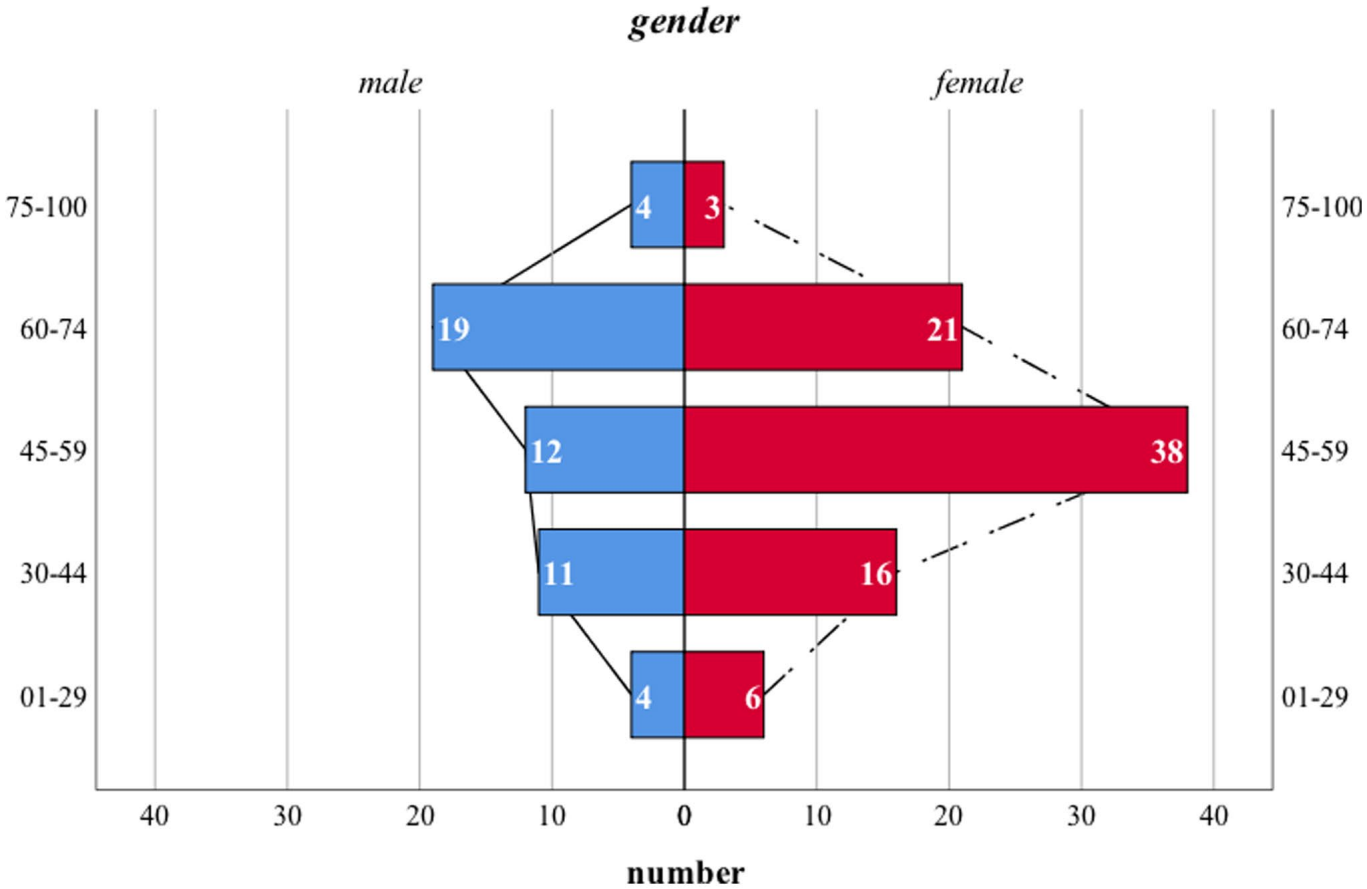
Age distribution pattern of patients with DM. The age pattern of either male or female patients was normally distributed. However, the mean age of initial diagnosis was higher in male patients. Notably, no statistical difference was noticed. *X*-axis: number of patients in each subgroup; *Y*-axis: 5 age intervals ranging from 1 to 29, 30 to 44, 45 to 59, 60 to 74, 75 to 100, respectively; blue columns: number of male patients; red columns: number of female patients; dash and solid black lines: reference line set for comparison.

Notably, the distribution pattern of the time of initial diagnosis among each gender varied. The most prevalent time interval in male patients was 60–75 while that of the females was 45–59 (Figure 2).

We also summarized the initial complaints of each individual and categorized them into nine subgroups (Supplementary Table S1). To emphasize, respiratory problems consist of dyspnea, cough, expectoration, etc., while unspecific skin lesions include overall erythema without specific locations, wheals, and purpura. All the other symptoms except for the listed items are allocated to the group “Others.” Overall, no statistical significance was noticed in either list of items. The prevalence of Gottron’s papules was far higher in MG than in either ATBG or NMG, but no statistical significance was noticed.

### Clinical features of malignancies

Clinical features of malignancies recorded in MG are illustrated in Table 2. Basically, 12 patients with MADM were documented, among whom 5 patients were proven anti-TIF1γ positive (41.67%).

**Table 2:** Characteristics of the malignancy group.

Patient ID in EMRS	Age at onset of DM (Y)	Gender	Tumor entity	Time gap between malignancy and DM (M)	MSAs & MAAs
14015340	46	Female	Breast	−13	Ro52
12532821	53	Male	Lung	1	Ro52
12787817	70	Male	Nasopharyngeal	11	TIF1γ
13912388	53	Female	Gastric	0	TIF1γ, Mi2β, PM-SCL100
13416964	65	Male	Colorectal	1	Mi2α, Mi2β
01275122	69	Male	Gastric	−120	TIF1γ, Mi2α, PM-SCL75
13163699	77	Female	Esophageal	−98	TIF1γ, Ro52
03375570	61	Female	Fallopian tube	0	Ro52
13052536	67	Male	Lung	9	MDA5
12967053	39	Female	Nasopharyngeal	0	TIF1γ, Ro52
07571818	68	Female	Thyroid	−87	Ro52
12041289	46	Female	Cervical	−35	Mi2α, Mi2β, PM-SCL75

ID, identification; EMRS, electronic medical record system; Y, years of life; M, months; MSA, myositis specific autoantibody; MAA, myositis associated autoantibody. A minus number in the column of “time gap between malignancy and DM” indicates the malignancy was reported prior to the diagnosis of DM.

Among the listed malignancies, gastrointestinal tumors accounted for the most (33.33%), followed by nasopharyngeal cancers and lung cancers. Of note, the four confirmed gastrointestinal malignancies comprised two gastric cancers, one esophageal cancer, and one colorectal carcinoma.

Two-thirds of the malignancies were initially diagnosed within an interval of 5 years (i.e., 2 years prior to the diagnosis of DM and 3 years beyond the diagnosis) covering the time-point of initial diagnosis of DM (Supplementary Figure S1). Of the malignancies, 41.67% (*n* = 5) were diagnosed shortly before or after the confirmation of DM within half a year and another 16.67% (*n* = 2) in the subsequent 1 year.

### Tumor biomarker profiles in ATBG

One to five anomalously expressed tumor biomarkers were observed in each individual from ATBG. Noticeably, 31 patients (31/57) displayed only one abnormal tumor biomarker, while patients with three or more anomalous tumor markers accounted for some 15%.

Frequencies of aberrantly expressed tumor biomarkers are compared in Supplementary Figure S2. Basically, CEA, CA211, CA125, and NSE ranked as the top four abnormally expressed tumor biomarkers. Notably, ectopic expression of CA242, CA199, and β-HCG were most unlikely to occur in patients with DM.

### Laboratory parameters

Laboratory characteristics are summarized in Table 1. Several significant differences were observed in parameters including diagnostic age, ferritin, sedimentation, LDH, lymphocyte counts, and NLR. However, no statistically significant differences were proven among CK, CK-MB, and β2-microglobulin.

As regards the MSAs, among the three groups the prevalence of anti-TIF1γ and anti-Mi2 autoantibodies demonstrated a significant difference with a *p* value of 0.016 and 0.007, respectively. Our findings failed to demonstrate any correlation between other autoantibodies, including anti-NXP2, and the tendency of malignancies. Of note, co-expression of MSAs (more than two positive MSAs detected in one patient) existed in our retrospective analysis with an overall incidence of 6.7% (9/134). However, there are no statistical significance concerning the co-expression incidence of MSAs among different groups (*p* = 0.265) (Table 1).

### Models predictive of malignancies in DM

After feature refinement, an MLR model as a dimension reduction algorithm was used to assess the predictive probability of the eight selected features (Table 3). It showed the estimates of multinomial logistic regression coefficient, *p*-value, and odds ratio for each category of the model. In this model study, NMG was used as a reference item for comparison, and the model formulas were as follows:


$$\begin{array}{l} \log\;\left(\mathrm{MG}/\mathrm{NMG}\right)=-3.06+0.058^{*}\;\mathrm{Diagnostic}\;\mathrm{age}-0.019^{*}\;\mathrm{ESR}\\ +0.003^{*}\;\mathrm{LDH}-2.233^{*}\;\mathrm{Lymphocyte counts}+0.048^{*}\;\mathrm{NLR}\\ +1.283^{*}\;\mathrm{Anti}-\mathrm{TIF}1\gamma -1.660^{*}\;\mathrm{Anti}-\mathrm{Mi2}+0.001^{*}\;\mathrm{Ferritin}. \end{array}$$

$$\begin{array}{l} \log\;\left(\mathrm{ATBG}/\mathrm{NMG}\right)=-3.98+0.043^{*}\;\mathrm{Diagnostic}\;\mathrm{age}+0.219^{*}\;\mathrm{ESR}\\ +0.002^{*}\;\mathrm{LDH}-0.097^{*}\;\mathrm{Lymphocyte counts}+0.046^{*}\;\mathrm{NLR}-0.523^{*}\;\\ \mathrm{Anti}-\mathrm{TIF}1\gamma -1.547^{*}\;\mathrm{Anti}-\mathrm{Mi2}+0.009^{*}\;\mathrm{Ferritin}. \end{array}$$

**Table 3:** Multivariable multinomial logistic regression for the selected features in MG and NMG versus NMG.

Class	Selected variables	OR	2.5% CI	97.5% CI	Wald. value	*p*-value	Std. error
MG	Diagnostic age	1.06	1.012	1.111	2.44	0.015**	0.02
	ESR	0.981	0.937	1.027	−0.81	0.419	0.02
	LDH	1.003	0.999	1.007	1.50	0.132	0.00
	Lymphocyte counts	0.107	0.026	0.448	−3.06	0.002**	0.73
	NLR	0.953	0.843	1.078	−0.76	0.445	0.06
	Anti-TIF1γ	3.606	0.58	22.435	1.38	0.169	0.93
	Anti-Mi2	5.26	0.495	55.902	1.38	0.169	1.21
	Ferritin	1.001	1	1.002	1.62	0.105	0.00
ATBG	Diagnostic age	1.044	1.013	1.077	2.77	0.006**	0.02
	ESR	1.022	1.004	1.041	2.41	0.016**	0.01
	LDH	1.002	0.999	1.005	1.58	0.113	0.00
	Lymphocyte counts	0.907	0.509	1.616	−0.33	0.741	0.29
	NLR	1.048	0.979	1.121	1.34	0.18	0.03
	Anti-TIF1γ	0.593	0.162	2.174	−0.79	0.43	0.66
	Anti-Mi2	0.213	0.032	1.424	−1.60	0.111	0.97
	Ferritin	1.001	1	1.002	1.56	0.119	0.00

OR, odds ratio; CI, confidence interval; ^*^p value < 0.05; ^**^p value < 0.01.

From the fitted MLR, the variables such as diagnostic age (*p* = 0.015) and lymphocyte counts (*p* = 0.002) were seen to be more significant to the model MG than to the NMG. It indicated that the MG population was older and had lower lymphocyte counts.

Similarly, diagnostic age (*p* = 0.006) and ESR (*p* = 0.016) were found to be more significant to the model ATBG than to the NMG, and the ATBG population was older with higher ESR.

Moreover, Supplementary Table S2 shows the MG population had higher Anti-Mi2 levels (*p* = 0.015) and lower lymphocyte counts (*p* = 0.007) than the ATBG population.

Therefore, the lower lymphocyte counts can be used as an MG-specific indicator, an older age can be used as a specific indicator of an MG population, while anti-Mi2 can be used to distinguish MG and NMG populations and ESR can be used to distinguish ATBG and MG populations.

**Figure 3:**
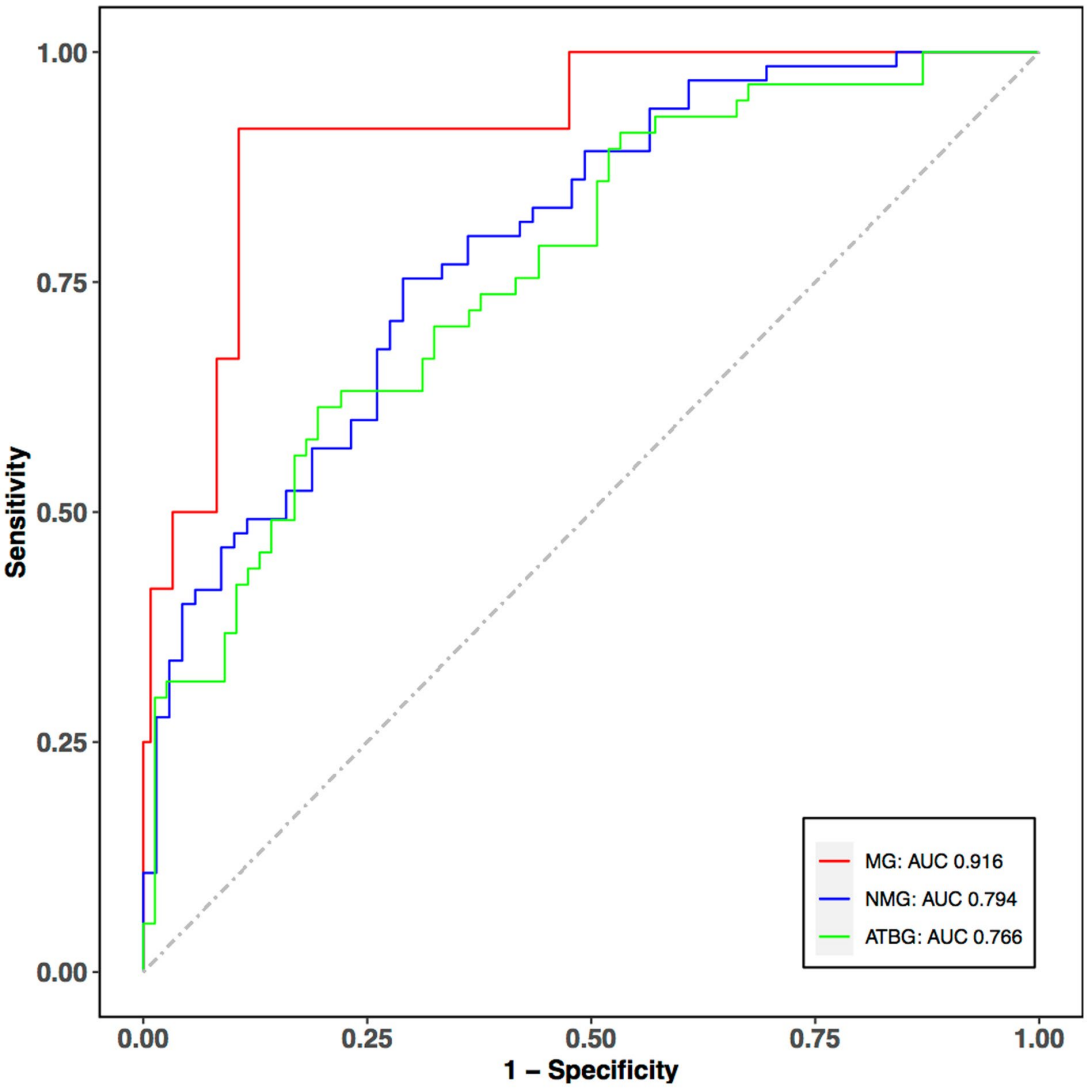
ROC curves of the classifier performance for the selected features. The red, blue, and green curves indicate the predictive performance of MG, NMG, and ATBG. The predictive power of MG and NMG group is fairly good. *X*-axis: 1-specificity; *Y*-axis: sensitivity; ROC, Receiver Operating Characteristic Curve; AUC, area under curve.

The selected features were used for subtypes classification by multivariable multinational logistic regression. Our model performance reached the average accuracy, F1-score, precision, recall, specificity, and AUC of 0.707, 0.624, 0.643, 0.604, 0.809, and 0.825, respectively. According to these results, the ROC curves are also provided (Figure 3). The ROC curves showed that AUC of the multiple logistic regression model were 0.916, 0.794, and 0.766 for MG, NMG, and ATBG, demonstrating that the model was a reasonable predictor for the discrimination of MADM.

## Discussion

In this study, we categorized a group in which the patients were diagnosed void of malignancies, but abnormal tumor biomarkers were documented. Series of retrospective descriptive analysis of MADM and tumor biomarkers expression profiles in ATBG were conducted. Moreover, risk factor analysis was implemented to reveal any possible differences from the Caucasian population. The incidence of malignancies in observed DM patients was 8.96%, of which digestive system cancers were shown to have the highest proportion. DM patients without malignancies were vulnerable to a wide spectrum of cancers, and the most commonly expressed abnormal tumor biomarkers include CEA, CA211, and CA125. In contrast to previous literature, no statistical significance of anti-NXP2 autoantibodies was found while anti-TIF1γ and anti-Mi2 autoantibodies were shown to be indicators of MADM. A powerful MLR model was established to predict the phenotype of dermatomyositis on the basis of whether a malignancy exists. The model indicates a positive correlation between age and malignancies risks.

MADM is deemed to be a large disease entity accounting for one-fifth of DM, while the exact percentage varies among different literature (11, 12). Compared to the general population, the pooled RR for patients with DM was 5.50 (4.31–6.67) (13). In our collective, 8.96% (12/134) of the patients had confirmed malignancies, which was similar to the overall malignancy prevalence (7.96%, 16/201) reported by another study recently conducted at Pennsylvania University (14). Basically, age has been perceived as a risk factor for MADM on the basis of real-world evidence and meta-analysis (1, 15, 16), indicating the importance of cancer screening in the elderly population. Recently, Zhong et al. established one nomogram, and the multivariate logistic analysis showed that age exceeding 50 years old was an independent risk factor for MADM (17). Nonetheless, Fardet et al. stated in their article that patients with MADM were prone to be diagnosed in a shorter period of time compared to those without tumors (18), which might be due to ethnic differences and divergent medical habits. To be specific, patients, particularly the senior group, are more inclined to ignore mild to moderate discomforts and are often reluctant to visit clinics or comprehensive hospitals.

Tumor location among MADM patients varies in relation to geographical distribution and ethnic group. In northern European countries, the dominant malignancies include cancers of ovaries, lung, pancreas, and gastrointestinal tract, and non-Hodgkin lymphoma, while in Asian countries, including Japan and Singapore, nasopharyngeal carcinoma, digestive system cancers, and lung cancers are more common (19–21). Unlike in the previously conducted research in central and southern China (22, 23), digestive system, nasopharyngeal, and lung malignancies were the most frequent cancers in eastern China. Several factors may contribute to this nuance including eating habits, climate, and living environment. Besides, most of the malignancies were proven shortly before or after the diagnosis of DM, which emphasizes the importance of tumor screening once DM is diagnosed in clinical practices.

The analyzed risk factors seemed contradictory to the previous reports. Herein, no statistical significance was noticed among all the chief complaints, while Gottron’s papules were postulated as a possible indicative index of MADM. Any conclusive role of cutaneous features in predicting malignancies has remained controversial. Fang and other scholars showed that patients with MADM were prone to display Gottron’s signs (24). However, Khanna et al. concluded that Gottron’s papules did not play either a positive or a negative role in predicting malignancies. Instead, Khanna considered Shawl sign as a predictive factor (25). Actually, the correlations between typical cutaneous signs and MADM have been widely studied. Muro et al. stated in one comprehensive review that several MSAs including Mi2 were relevant to Gottron’s papules (26), indicating a potential intermediary role of MSAs in clinical features and DM.

Our findings suggest that although anti-TIF1γ and anti-Mi2 autoantibodies were also proven indicators of MADM, no correlation between anti-NXP2 autoantibody and the occurrence of tumors was found. Basically, the clinical significance of MSAs has been well accepted in recent practices. For instance, anti-TIF1γ autoantibody, identified in 7 ~ 31% of adult patients with DM, was proven as an indicator of MADM (27). Of DM patients with positive anti-TIF1-antibodies, 58% are prone to suffer from solid or hematological tumors with an OR of 27.26 (28). However, the association between anti-NXP2 autoantibody and cancer is mild (29). Fiorentino and Bowerman proposed that anti-NXP2 antibody was strongly associated with cancer while two other studies, together with our work, did not confirm any possible correlation (14, 30–32). The contradictory findings highlight the need for further studies on the clinical feature and underlying mechanism of anti-NXP2. Of note, given that the studies focusing on the indicative role in MADM of anti-Mi2 autoantibody are limited (33), our work provides further concrete evidence. Interestingly, co-expressions of MSAs are documented in our retrospective work although the incidence remains quite low (9/134); no statistical significance was noticed among different groups.

Tumor biomarker profiles from ATBG were analyzed. The majority of anomalously expressed tumor biomarkers include CEA, CA211, CA125, and NSE. CEA and CA211 strongly indicate digestive system carcinoma including gastric carcinoma, rectal tumors, and esophageal malignancies, while CA125 indicates either ovarian or digestive system carcinoma (34–37). NSE is currently the most reliable tumor marker in diagnosis, prognosis, and follow-up of small cell lung cancer as the level of NSE correlates with tumor burden, conditions of metastasis, and treatment response (38). These findings indicate that DM patients with abnormal tumor biomarkers are vulnerable to suffering from malignancies similar to those seen in MG. Most importantly, we simulated a comprehensive model which is highly potent and effective in predicating malignancies in patients with DM. Long-term follow-ups and regular targeted cancer screening are of great significance in such a population.

Of note, DM displays a bimodal age distribution (5), but owing to administration policy, no pediatric patients are hospitalized, making the other peak unnoticeable. The sample size of the study is relatively small and all the clinical data derive from a single center which may lead to some bias pertaining to the final conclusion.

To conclude, this study firstly categorized patients into three groups according to malignancies and tumor biomarkers, and thereafter compared the intergroup phenotypes of MADM in one single center located in eastern China and suggested that anti-TIF1γ and anti-Mi2 autoantibodies may be useful predictors. Furthermore, one MLR model with sufficient capacity was established to predict the phenotypes of DM. Our study also highlighted the importance of regular follow-ups in DM patients with abnormal tumor biomarkers.

## Data availability statement

The original contributions presented in the study are included in the article/Supplementary material, further inquiries can be directed to the corresponding author.

## Ethics statement

The studies involving human participants were reviewed and approved by Second Affiliated Hospital, School of Medicine, Zhejiang University. Written informed consent for participation was not required for this study in accordance with the national legislation and the institutional requirements.

## Author contributions

X-YM contributed to conception and design of the study. Z-LT and C-CC organized the database. C-CC applied for IRB permission. Z-LT performed the statistical analysis and wrote the first draft of the manuscript. Z-WT and X-WL revised sections of the manuscript. All authors contributed to the article and approved the submitted version.

## Conflict of interest

The authors declare that the research was conducted in the absence of any commercial or financial relationships that could be construed as a potential conflict of interest.

## Publisher’s note

All claims expressed in this article are solely those of the authors and do not necessarily represent those of their affiliated organizations, or those of the publisher, the editors and the reviewers. Any product that may be evaluated in this article, or claim that may be made by its manufacturer, is not guaranteed or endorsed by the publisher.
