# Supplementary material for: Malignancy in dermatomyositis: a mono-centric retrospective study of 134 patients in China and a potential predictive model
Source: Front Med (Lausanne). 2023 Jun 8;10:1200804. doi: 10.3389/fmed.2023.1200804 (PMC10285222; doi:10.3389/fmed.2023.1200804)
Supplement: Supplementary file 1 [file Data_Sheet_1.docx]

**Supplementary Figures and tables**

**Supplementary Figure 1 Time gap between the onset of malignancies and DM**

Most of the malignancies were confirmed within three years prior to or posterior to the diagnosis of DM. X-axis: time in months, the time-point 0 months represents the co-occurrence of the initial diagnoses of DM and malignancy. Y-axis: frequency of malignancies in each time interval.

**Supplementary Figure 2 Frequency of aberrantly expressed tumor biomarkers**

CEA, CA211, CA125 and NSE are the most documented aberrantly expressed tumor biomarkers. X-axis: detected abnormally expressed tumor biomarkers. Y-axis: frequency of the records.

**Supplementary Table 1 Initial complaints or signs prior to the confirmation of DM**

|  | MG | ATBG | NMG | *p* value | Significance |
| --- | --- | --- | --- | --- | --- |
| Heliotrope rash | 3(25%) | 10(17.54%) | 15(23.08%) | 0.706 | ns |
| Gottron’s papules | 3(25%) | 4(7.02%) | 4(6.15%) | 0.084 | ns |
| Muscle Weakness | 3(25%) | 12(21.05%) | 15(23.08%) | 0.940 | ns |
| Arthritis or arthrocele | 2(16.67%) | 9(15.79%) | 4(6.15%) | 0.199 | ns |
| Dryness | 0(0%) | 2(3.51%) | 0(0%) | 0.254 | ns |
| Respiratory problems | 0(0%) | 10(17.54%) | 10(15.38%) | 0.298 | ns |
| Unspecific skin lesions | 6(50%) | 20(35.08%) | 30(46.15%) | 0.388 | ns |
| Raynaud syndrome | 0(0%) | 2(3.51%) | 1(1.54%) | 0.657 | ns |
| Others | 0(0%) | 4(7.02%) | 4(6.15%) | 0.645 | ns |

Abbr.: MG, malignancy group; ATBG, aberrant tumor-biomarker group; NMG, non-malignancy group; ns, not significant

Comparison of initial complaints and signs were conducted but no statistical difference was noticed in either item.

**Supplementary Table 2 Multivariable multinomial logistic regression for the selected features in MG and NMG versus AGBT.**

| **Class** | **Selected Variables** | **OR** | **2.5% CI** | **97.5% CI** | **Wald. Value** | **p.value** | **std.error** |
| --- | --- | --- | --- | --- | --- | --- | --- |
| MG | Diagnostic age | 1.015 | 0.951 | 1.083 | 0.456 | 0.648 | 0.033 |
|  | ESR | 0.96 | 0.916 | 1.006 | -1.698 | 0.089 | 0.024 |
|  | LDH | 1.001 | 0.997 | 1.004 | 0.303 | 0.762 | 0.002 |
|  | Lymphocyte counts | 0.118 | 0.025 | 0.551 | -2.719 | 0.007 ** | 0.785 |
|  | NLR | 0.91 | 0.797 | 1.039 | -1.391 | 0.164 | 0.068 |
|  | Anti-TIF1γ | 6.085 | 0.92 | 40.25 | 1.873 | 0.061 | 0.964 |
|  | Anti-Mi2 | 24.728 | 1.884 | 324.487 | 2.442 | 0.015 ** | 1.313 |
|  | Ferritin | 1.015 | 0.999 | 1.001 | 0.505 | 0.614 | 0.000 |
| NMG | Diagnostic age | 0.958 | 0.928 | 0.989 | -2.667 | 0.008 ** | 0.016 |
|  | ESR | 0.978 | 0.961 | 0.996 | -2.406 | 0.016 ** | 0.009 |
|  | LDH | 0.998 | 0.995 | 1.001 | -1.550 | 0.121 | 0.002 |
|  | Lymphocyte counts | 1.102 | 0.614 | 1.98 | 0.326 | 0.745 | 0.299 |
|  | NLR | 0.955 | 0.891 | 1.023 | -1.312 | 0.190 | 0.035 |
|  | Anti-TIF1γ | 1.687 | 0.46 | 6.191 | 0.789 | 0.430 | 0.663 |
|  | Anti-Mi2 | 4.7 | 0.698 | 31.638 | 1.591 | 0.112 | 0.973 |
|  | Ferritin | 0.999 | 0.998 | 1 | -1.559 | 0.119 | 0.001 |

Abbr.: OR, Odds Ratio; CI, confidence interval;

^*^_,_ *p* value <0.05; ^**^_,_ *p* value <0.01.
