# Supplementary figures and images for: Malignancy in dermatomyositis: a mono-centric retrospective study of 134 patients in China and a potential predictive model
Source: Front Med (Lausanne). 2023 Jun 8;10:1200804. doi: 10.3389/fmed.2023.1200804 (PMC10285222; doi:10.3389/fmed.2023.1200804)

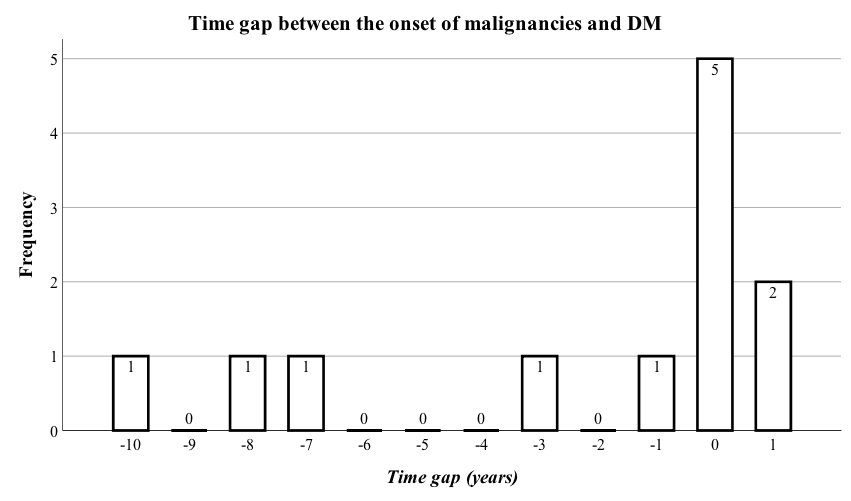

Supplement: Supplementary file 2 [file Image_1.TIF]

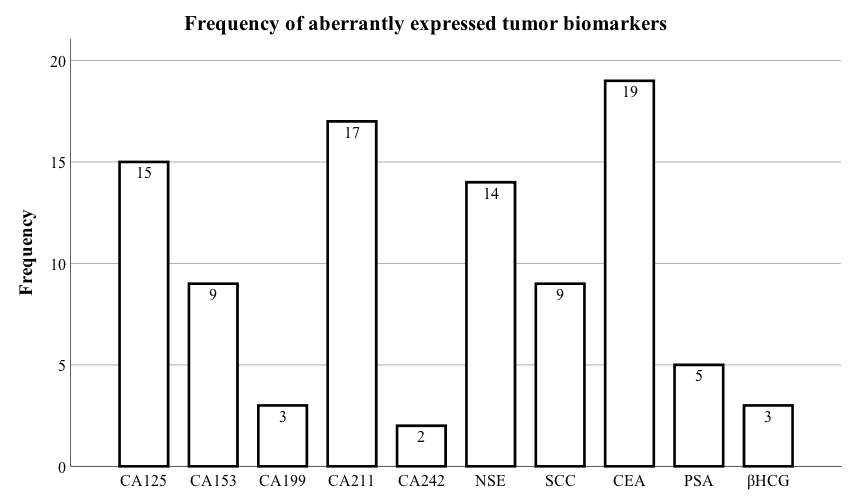

Supplement: Supplementary file 3 [file Image_2.TIF]
